# Bamboo Pulp Toughening Poly (Lactic Acid) Composite Using Reactive Epoxy Resin

**DOI:** 10.3390/polym15183789

**Published:** 2023-09-17

**Authors:** Krittameth Kiattipornpithak, Pornchai Rachtanapun, Sarinthip Thanakkasaranee, Pensak Jantrawut, Warintorn Ruksiriwanich, Sarana Rose Sommano, Noppol Leksawasdi, Thorsak Kittikorn, Kittisak Jantanasakulwong

**Affiliations:** 1 School of Agro-Industry, Faculty of Agro-Industry, Chiang Mai University, Mae-Hea, Mueang, Chiang Mai 50100, Thailand; first200294@gmail.com (K.K.); pornchai.r@cmu.ac.th (P.R.); sarinthip.t@cmu.ac.th (S.T.); noppol.l@cmu.ac.th (N.L.); 2 Cluster of Agro Bio-Circular-Green Industry, Faculty of Agro-Industry, Chiang Mai University, Chiang Mai 50100, Thailand; pensak.amuamu@gmail.com (P.J.); warintorn.ruksiri@cmu.ac.th (W.R.); 3 Department of Pharmaceutical Sciences, Faculty of Pharmacy, Chiang Mai University, Muang, Chiang Mai 50200, Thailand; 4 Faculty of Agriculture, Chiang Mai University, Chiang Mai 50200, Thailand; sarana.s@cmu.ac.th; 5 Division of Physical Science, Faculty of Science, Prince of Songkla University, Songkhla 90110, Thailand; thorsak.k@psu.ac.th

**Keywords:** poly (lactic acid), bamboo pulp, epoxy resin, reaction, reactive melt blending, reactive into cross-linking reaction

## Abstract

A novel poly (lactic acid) (PLA) composite with excellent mechanical properties, toughness, thermal stability, and water resistance was developed using a reactive melt-blending technique. PLA was melt mixed with epoxy resin (EPOXY) and bamboo pulp (PULP) to improve its reaction and mechanical properties. FTIR analysis confirmed the successful reaction of the PLA/EPOXY/PULP composites; the epoxy groups of EPOXY reacted with the –COOH groups of PLA and the –OH groups of PULP. The PLA/EPOXY/PULP5 composite showed a high tensile strength (67 MPa) and high toughness of 762 folding cycles, whereas the highest tensile strength was 77 MPa in the PLA/EPOXY5/PULP20 sample. SEM images presented a gap between the PLA and PULP; gap size decreased with the addition of EPOXY. The T_g_ of the PLA decreased with the EPOXY plasticizer effect, whereas the T_m_ did not significantly change. PULP induced crystallinity and increased Vicat softening of the PLA/PULP and PLA/EPOXY/PULP composites. The EPOXY reaction of the PLA/PULP composites improved their tensile properties, toughness, thermal stability, and water resistance.

## 1. Introduction

Natural fibers are used to reinforce polymers, reduce polymer consumption, and increase the degradation ability of polymers. They are biodegradable, lightweight, inexpensive, non-toxic, recyclable, and possess good mechanical properties. Moreover, natural fibers are used as load bearings in polymers [1]. The main structure of natural fibers is cellulose, which is obtained from plants. Bacteria can be used to produce bacterial cellulose [2]. Various plants (cotton [3], ginger [4], rice straw [5], or bamboo [6]) and bacteria (*Acetobacter xylinum* [2]) are used to produce cellulose. Cellulose and bacterial cellulose have excellent mechanical and thermal properties with high crystal and surface area, enabling it to have wide applications.

Bamboo is a fast-growing and readily available plant in Southeast Asia that is commonly used as a natural fiber [7]. Bamboo fibers are produced from raw bamboo using mechanical and chemical pulping processes [7]. To obtain pure cellulose from bamboo fibers, an extraction technique is employed to remove lignin and hemicellulose. Lignin, present in the fibers, serves as a binder for fiber reinforcement due to its ability to chemically react with hydrophilic polymers [8]. However, some lignin in the fiber is lost during the pulping process, decreasing the interfacial adhesion between the microfibrils and the mechanical properties of the polymer composite [9,10,11].

Poly (lactic acid) (PLA) is a popular biodegradable polymer used in various industries such as packaging, agricultural, and medical industries [12]. PLA, however, has certain disadvantages such as brittleness, low impact resistance, high cost, and low thermal resistance, which limit its applications. Packaging from the PLA injection molding process breaks easily from its brittleness, while high temperature (>60 °C) induces its shape distortion. Various additives, such as poly (glycolic acid) (PGA), epoxy, and poly (hydroxykanoates) (PHA), have been studied to enhance the flexibility of PLA [13,14,15]. In addition, natural fiber reinforcement is used in PLA to improve its mechanical properties and enhance its thermal stability.

Epoxy resin (EPOXY) is a possible crosslinking agent that induces reactions and interactions between the reactive functional groups of the fiber and PLA. EPOXY contains epoxy groups in its main structure, acting as crosslinking agents for various reactive functional groups such as hydroxyl, carboxylic, and amide groups [16]. Bisphenol A, a precursor commonly used in the synthesis of epoxy resins, is produced from alcohols, thiols, and amines by adding epichlorohydrin [16]. EPOXY is used to improve the properties of materials, including toughness, heat resistance, shrinkage, and corrosion resistance [17,18,19,20]. Reactions of the epoxy groups of EPOXY with carboxylic [21], hydroxyl [21], and amine [22] groups have been reported. Reactive blending of polymers is an effective method for improving the mechanical and thermal properties, morphology, water resistance, and reaction of the blend [23,24]. The reactions of the epoxy groups influence the thermal, morphological, and crystal behaviors of the polymer blend [25]. The addition of EPOXY to the PLA matrix enhances the branched structures, crosslinking network, and molecular weight [26]. Crosslinking has been reported to enhance the network structure and properties of polymers [27,28]. Moreover, using epoxy as a compatibilizer has been reported to enhance the compatibility of PLA and nylon blends [29]. EPOXY has been reported to improve the properties of PLA [30,31,32]. However, improvement in the properties of PLA/bamboo fibers by the addition of EPOXY to increase the interfacial adhesion between PLA and the fibers has not been investigated in detail. EPOXY is a chain extender for PLA and a compatibilizer to improve compatibility between PLA and fiber composite which improve mechanical properties, toughness, thermal stability, and water resistance. Therefore, deep detail of reaction mechanism effect to PLA/fiber composite via reactive compatibilizer, is the key to study and improve its properties for wide applications.

In this study, bamboo pulp was mixed with PLA and EPOXY to induce chemical reactions and improve the composite properties. Bamboo pulp was utilized as a reinforcement for PLA, while EPOXY was employed as a crosslinking agent to establish crosslinks between PLA and the bamboo fibers. The mechanical properties, folding cycles, morphology, water resistance, heat resistance, and chemical reactions of the resulting composite were investigated.

## 2. Materials and Methods

### 2.1. Materials

Polylactic acid 4032D (PLA) pellets were purchased from NatureWorks LLC (Minnetonka, MN, USA) at a density of 1.24 g/cm^3^, MW 100,000 g/mol, MFI 7 g/10 min at 210 °C. Epoxy resin (Diglycidyl ether of bisphenol A) grade 0302 was purchased from EASY Resin Co., Ltd. Nonthaburi, Thailand. Bamboo pulp (Bambusa nutans Wall.) was purchased from Chiang Mai Province, Thailand. Sodium hydroxide (NaOH) analytical reagent grade was purchased from vs. chem house Ltd., Bangkok, Thailand.

### 2.2. Bamboo Pulp Preparation

Bamboo pulp was prepared without bleaching process to reduce the strong chemical treatment process. The bamboo chips were dried in a hot air oven at 80 °C for 48 h, pretreated with NaOH (18 wt.%) aqueous solution at the pulp ratio of 1:20, and boiled at 85 °C for 3 h to eliminate the residual lignin, fatty acid, hemicellulose, and other impurities. The samples were then dissolved in distilled water and dried in an oven. Then, the dried samples (sizes lower than 250 microns) were sieved.

### 2.3. Sample Preparation

**Figure 1:**
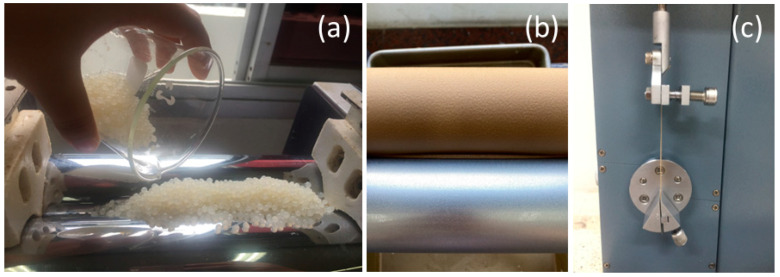
Images of (**a**) two-roll mills melt mixing, (**b**) PLA/EPOXY/PULP blend sample, and (**c**) folding cycles test.

The PLA was melt blended with epoxy resin using two-roll mills (Figure 1a,b) with a speed of 50 rpm at 160 °C for 5 min; then, bamboo pulp and epoxy were added, respectively, and mixed for 15 min. The samples were compressed into sheets using hot compression at 160 °C for 10 min, followed by quenching in cool water. The compositions and code names of PLA, EPOXY, and PULP in the composites are listed in Table 1.

### 2.4. Fourier-Transform Infrared Spectroscopy (FTIR)

The reaction mechanism was studied using ATR method by Fourier-transform infrared spectroscopy (FTIR/IR–4700, Jasco Corp., Tokyo, Japan). The samples for the infrared spectra (FTIR) were prepared into thin films with a thickness of approximately 100 µm. The IR spectra were measured from 4000 to 500 cm^−1^ with 4 cm^−1^ of resolution.

### 2.5. Tensile Properties

The tensile strength and elongation at break were determined using a mechanical testing machine (MCT–1150; A&D Company Limited, Tokyo, Japan). The shape of the dog-bone test was prepared according to JIS K 6251–7. The sample sheets were prepared with dimensions (width, length, and thickness) of 5 mm, 30 mm, and 1 mm, respectively. The samples were kept at 25 °C with 50 ± 2% RH for 24 h. Samples were derived by averaging five experimental runs for each sample.

### 2.6. Differential Scanning Calorimetry (DSC)

The thermal analysis was performed using a differential calorimeter (DSC 823E; Mettler Toledo, OH, USA). Approximately 5–10 mg of each sample was placed in a closed aluminum pan. Differential scanning calorimetry (DSC) analysis was performed from 0 to 200 °C at a heating and cooling rate of 10 °C min^−1^.

### 2.7. Vicat Softening Temperature (VST)

Softening point measurements were performed using Vicat apparatus (c-090), Kijwisai, Bangkok, Thailand with a modified technique that allowed the standard ASTM D1525. The dimensions of the square test samples were 10 × 10 × 3 mm. All samples were derived by averaging five experimental runs for each sample.

### 2.8. Contact Angle

Contact angles were observed using a DSA30E Krüss GmbH instrument (Hamburg, Germany). Drop shape analysis was used to monitor the water contact angle. Water was dropped on the film surface and images were captured automatically every 20 s for 10 min.

### 2.9. Scanning Electron Microscopy (SEM)

The morphology was examined using scanning electron microscopy (SEM, JEOL JSM–5910LV JEOL Co., Ltd., Tokyo, Japan) with a magnification of 2000× at 15 KV. The samples were broken in liquid nitrogen followed by coating with a thin layer of gold.

### 2.10. Folding Endurance

Folding cycles were determined using Gotech GT 6014 A (Gotech Testing Machines, Inc., Taichung, Taiwan). The dimensions of the rectangular test samples were 100 × 15 × 1 mm^3^. A grip clearance of 10 mm was used (Figure 1(c)). All samples were conditioned and derived by averaging five experimental runs for each sample.

### 2.11. Statistical Analysis

To analyze the results, one-way ANOVA was performed using the Statistical Package for Social Sciences (SPSS Version 17, Armonk, NY, USA). Differences (*p* < 0.05) were evaluated using Duncan’s test.

## 3. Results and Discussion

### 3.1. Reaction Mechanism

**Figure 2:**
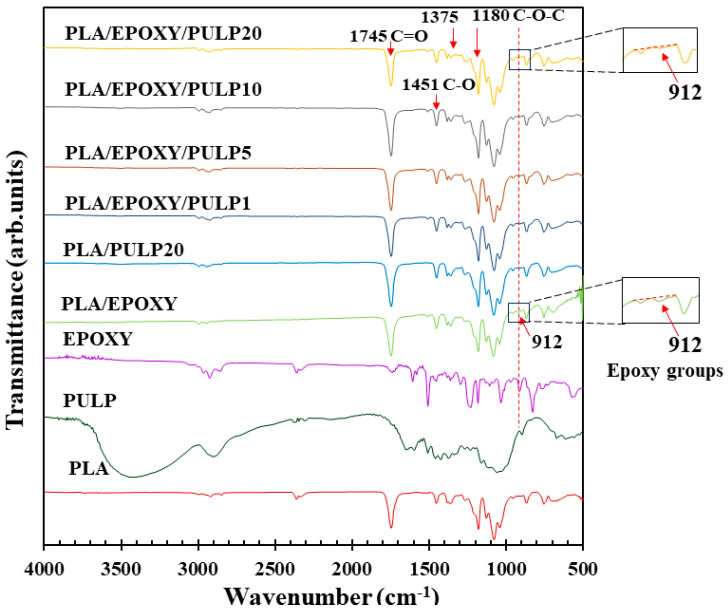
FTIR spectra of PLA, PULP, EPOXY, PLA/PULP, and PLA/EPOXY blend with PULP 1–20%.

**Figure 3:**
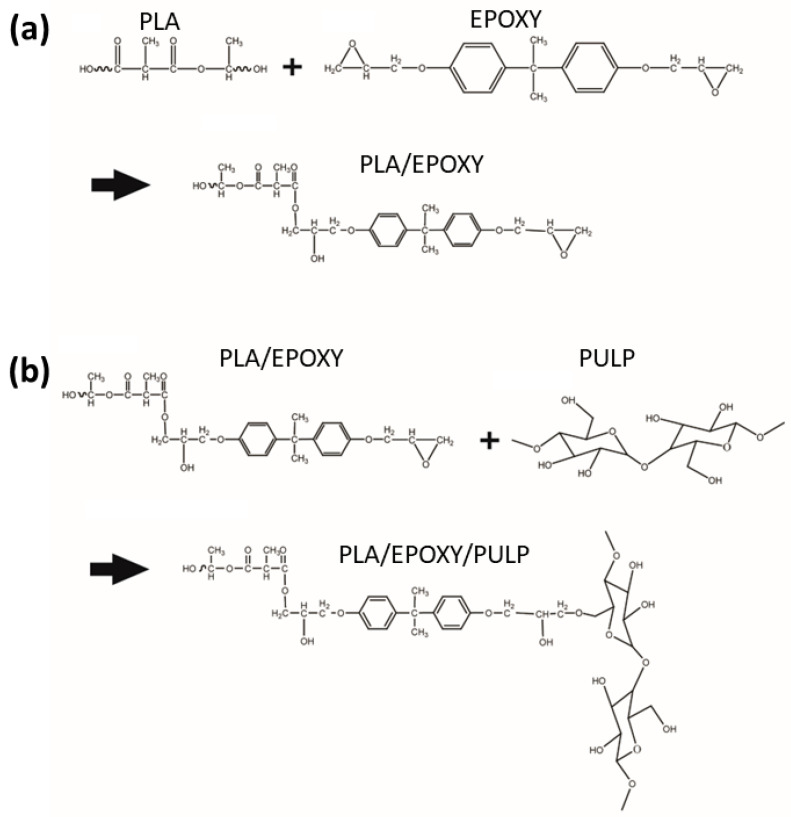
Expected reactions of (**a**) PLA/EPOXY and (**b**) PLA/EPOXY/PULP.

FTIR spectra of PLA, PLA/EPOXY, and PLA/EPOXY/PULP 1–20% are shown in Figure 2. PLA showed peaks at 1748 cm^−1^, 1451 cm^−1^, and 1180–955 cm^−1^ owing to the stretching vibrations of C=O (ester carbonyl), C–H, and C–O–C, respectively [33]. EPOXY presented a sharp peak of asymmetric vibrations, an aromatic oxirane ring at 912 cm^−1^, aromatic C=C stretching vibrations at 1509 and 1608 cm^−1^, and aromatic C–H vibrations of the bisphenol A moiety in the hyperbranched epoxy resin at 3059 cm^−1^ [15]. PLA/PULP20 presented C–H peaks at 1451 and 1378 cm^−1^, a stretching vibration of C=O at 1748 cm^−1^, absorption peaks of C–O–C at 1086 cm^−1^ and 1178 cm^−1^, and –OH peaks at 3500–4000 cm^−1^ [34]. The PLA/EPOXY composite exhibited characteristic peaks corresponding to the aromatic C=C stretching at 1509 cm^−1^ and the aromatic oxirane ring of EPOXY at 912 cm^−1^. EPOXY reacted with the carboxylic groups, and the reaction between the epoxy groups of EPOXY and the –COOH groups of PLA had been confirmed in a previous study [14]. The PLA/EPOXY/PULP samples showed combined spectra of PLA, EPOXY, and PULP, exhibiting the O–H peak of PULP at 3500–4000 cm^−1^, C=O stretching of PLA at 1748 cm^−1^, a C–H peak of PLA at 1451 cm^−1^, aromatic C=C stretching of EPOXY at 1509 cm^−1^, and an aromatic oxirane ring at 912 cm^−1^. The intensity of the aromatic oxirane ring peak at 912 cm^−1^ in the PLA/EPOXY/PULP samples was lower than that in the PLA/EPOXY sample. This decrease indicated the reduction of aromatic epoxy groups, ring opening of epoxy groups, and reacting with OH groups of PULP. Reactions between the epoxy and –OH groups have also been reported [35]. Hence, EPOXY reacted with both the –COOH groups of PLA and the –OH groups of PULP, connecting PLA with the PULP structures. The expected reactions are shown in Figure 3.

### 3.2. Mechanical Properties

**Figure 4:**
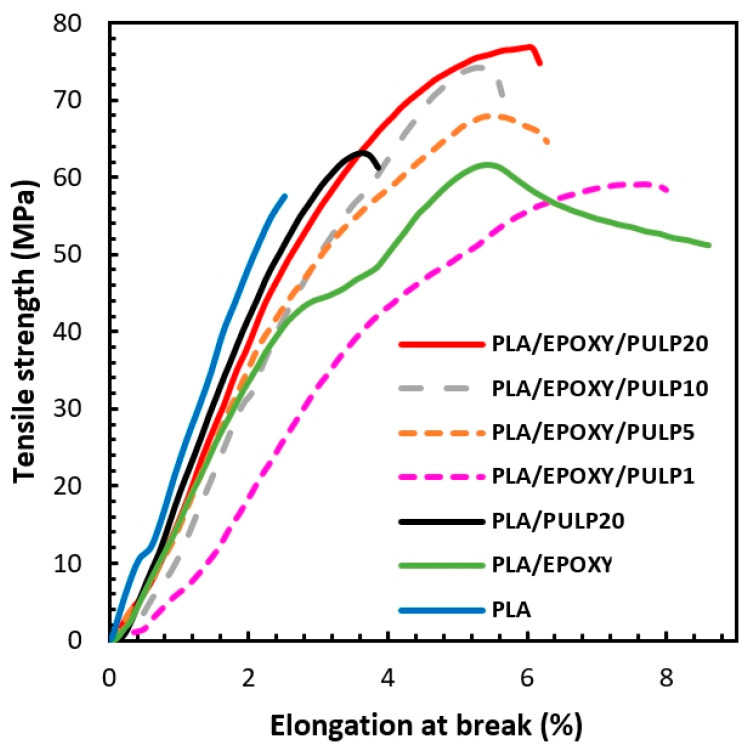
Stress–strain curves of PLA, PLA/EPOXY, PLA/PULP20, and PLA/EPOXY with PULP 1–20% composites.

The tensile strengths and elongations at the break of the PLA, PLA/EPOXY, PLA/PULP, and PLA/EPOXY/PULP composites are shown in Figure 4. PLA showed tensile strength and elongation at a break of 57 MPa and 2%, respectively. Tensile strength (50–60 MPa) and elongation at a break (2–6%) of pure PLA (4032D) have been previously reported [14,36,37]. Tensile strength and elongation at break of PLA composites with henequen (60 MPa, 0.7%) [38], bamboo (32.5 MPa, 3.2%) [6], and bagasse (55 MPa, 1.5%) [39] fibers, respectively, have also been reported. The incorporation of EPOXY into PLA resulted in an increase in tensile strength (62 MPa) and elongation at break (8%) compared to pure PLA, attributed to the crosslinking reaction that occurred inside the PLA matrix through the reaction with EPOXY [14]. The PLA/PULP20 sample exhibited 4 MPa and 3.5% tensile strength and elongation at break, respectively. The tensile strength of the PLA/EPOXY/PULP composites increased with increasing pulp content, whereas the elongation at break decreased compared with that of the PLA/EPOXY blend. The tensile strength of PLA/EPOXY/PULP20 reached a maximum of 76.8 MPa, 28% higher than pure PLA. The PLA/EPOXY5/PULP20 composite showed an elongation at break of 6% owing to the crosslinking provided by EPOXY within the PLA matrix and at the interface with the bamboo pulp fibers. This interfacial crosslink transferred strength through the bamboo pulp, reinforcing the PLA matrix. The bamboo pulp acted as a reinforcing fiber for the PLA matrix, whereas the EPOXY provided a crosslinking network. The combination of crosslinking inside PLA via the EPOXY reaction, interfacial crosslinking between PLA and pulp via the EPOXY reaction, and bamboo pulp-reinforcing PLA improved the tensile properties of the composites. Hence, EPOXY improves the mechanical properties of PLA by crosslinking, and plasticizer effects have been previously reported [8,11,40,41,42].

### 3.3. Folding Endurance

**Figure 5:**
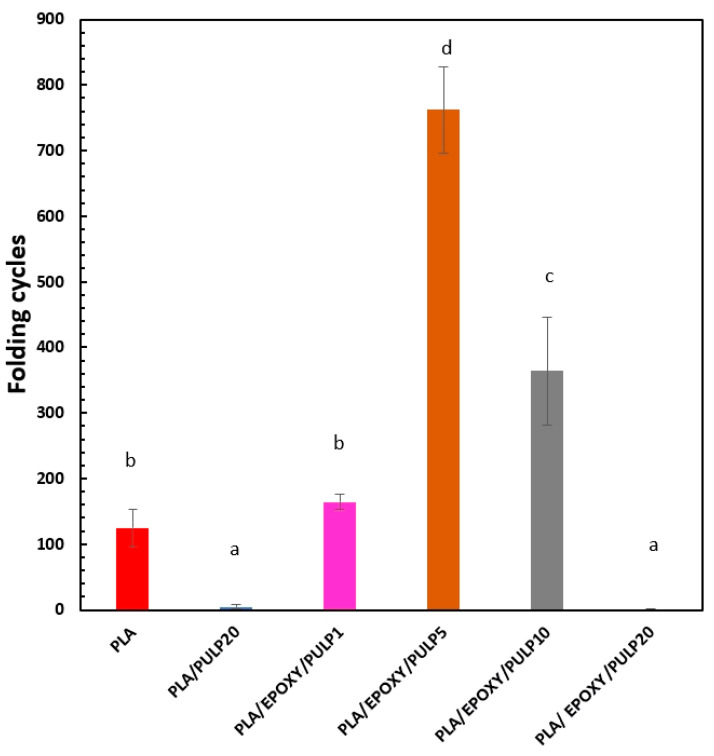
Folding cycles of PLA, PLA/PULP20, and PLA/EPOXY/PULP composites with 1–20% bamboo pulp content. The mean values indicated by the different lowercase letters significantly differ (*p* < 0.05).

Folding endurance is the most useful index of the bending film test, crucial to evaluating the toughness of a sample. Hence, this method was used to clarify the relationship between the folding test results and the mechanical properties of the films. Figure 5 shows folding tests of PLA, PLA/PULP20, and PLA/EPOXY/PULP composites with 1–20% bamboo pulp. The folding cycles for pure PLA, PLA/PULP20, PLA/EPOXY/PULP1, PLA/EPOXY5/PULP5, PLA/EPOXY5/PULP10, and PLA/EPOXY5/PULP20 were 124, 4, 164, 762, 364, and 1 cycle, respectively. The addition of pulp and EPOXY to PLA increased the number of folding cycles because EPOXY reacted with the carboxylic acid groups of PLA and the OH groups of the bamboo pulp [35], extending the PLA chain and inducing crosslinking in the PLA phase. EPOXY acted as both a crosslinking agent and a plasticizer, resulting in increased tensile strength and improved flexibility of PLA [14]. Moreover, EPOXY enhanced interfacial adhesion and decreased the number of voids between PLA and bamboo pulp. The incorporation of EPOXY in the PLA/EPOXY5/PULP5 composite resulted in decreased cracking, improved toughness, and increased folding cycles (764 cycles), demonstrating the beneficial reaction and plasticizer effects of EPOXY. However, adding 20% pulp in PLA negatively affected the folding cycle owing to the hardness of the bamboo pulp, leading to increased brittleness in the sample [43].

### 3.4. Morphology

**Figure 6:**
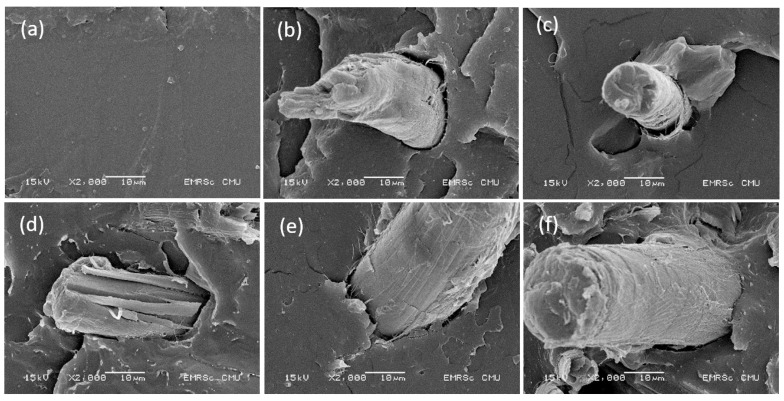
SEM images of (**a**) PLA, (**b**) PLA/PULP20, (**c**) PLA/EPOXY/PULP1, (**d**) PLA/EPOXY/PULP5, (**e**) PLA/EPOXY/PULP10, and (**f**) PLA/EPOXY/PULP20.

Figure 6 shows the morphologies of the PLA/EPOXY, PLA/PULP, and PLA/PULP/EPOXY composites. The PLA image in Figure 6a presents a smooth fracture surface. PLA/PULP exhibited dispersed PULP in the PLA matrix (Figure 6b). An incompatible blend of PLA and PULP with a large surface area was observed owing to the weak interfacial adhesion between PLA and the bamboo pulp, providing low elongation at break. The micrographs of PLA with bamboo pulp and the EPOXY composite showed a smaller interfacial space between PLA and bamboo pulp compared to PLA/PULP20, owing to the improvement of interfacial adhesion via a chemical reaction of EPOXY. The EPOXY reaction acted as a bridge and interfacial crosslinking between the terminal carboxyl groups of PLA and the hydroxyl groups of the bamboo pulp, improving the mechanical properties of the samples. The EPOXY reaction improved the morphology and mechanical properties of the PLA, as has been previously reported [42,44].

### 3.5. Thermal Properties

**Figure 7:**
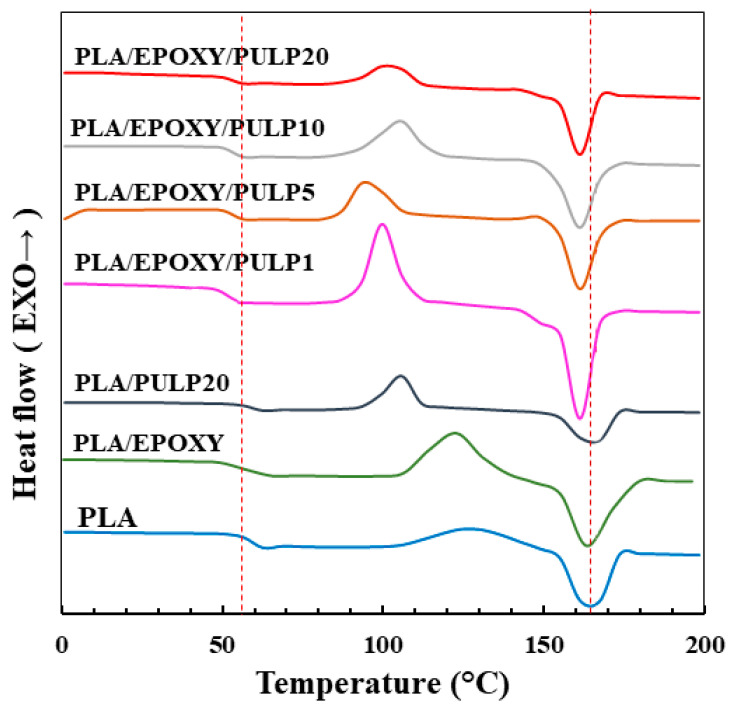
Second scan DSC thermograms of PLA, PLA/EPOXY, PLA/PULP, and PLA/EPOXY with 1–20% bamboo pulp content.

The thermal properties of PLA, PLA/EPOXY, PLA/PULP, and PLA/EPOXY/PULP composites with different bamboo pulp content (0–20%) were investigated using DSC. DSC 2nd scan was performed to obtain the crystallinity, T_m_, T_c_, and T_g_ of the samples. Figure 7 shows the 2nd scan DSC thermograms of PLA, PLA/EPOXY, PLA/PULP, and PLA/EPOXY with 1–20% bamboo pulp content. The T_g_ and T_m_ of PLA were 56 °C and 163 °C, respectively. PLA/PULP presented T_g_ and T_m_ that were almost identical to those of pure PLA. Adding EPOXY in PLA and PLA/PULP reduced the T_g_ of PLA from 56 °C to 51 °C (Table 2) owing to the plasticizer effect of EPOXY [14]. Adding EPOXY in PLA/PULP reduced the T_m_ of PLA from 163 °C to 161 °C owing to smaller crystal formation than pure PLA. The low T_c_ of the PLA/PULP and PLA/EPOXY/PULP composites compared to those of PLA and PLA/EPOXY was attributed to the small cold-crystal size formed by the nucleating effect of the bamboo pulp. The crystallinities of pure PLA, PLA/EPOXY, and PLA/PULP20 were 9.4%, 3.6%, and 15.2%, respectively (Table 2). The reduction in the number of PLA crystals in PLA/EPOXY can be attributed to the crystal obstruction caused by the crosslinking and plasticizer effects of EPOXY. The PLA/EPOXY/PULP composites showed a crystallinity of 7.4–18.8% owing to the combined nucleating agent effect of bamboo pulp and the crystal obstruction effect of EPOXY crosslinking in the PLA phase. The low crystal degree of PLA/EPOXY/PULP1 (7.4%) was due to the low movement of the PLA chain to from crystal by EPOXY reaction and plasticizer obstruction effect of excess epoxy in PLA. The addition of PULP 5–20% in PLA/EPOXY enhanced the degree of crystallization of PLA to 17–18% compared to PLA/EPOXY/PULP1 (7.4%) because the high amount of pulp and the rough surface of pulp induced nuclei of crystal formation as a nucleating agent.

### 3.6. Vicat Softening Temperature (VST)

**Figure 8:**
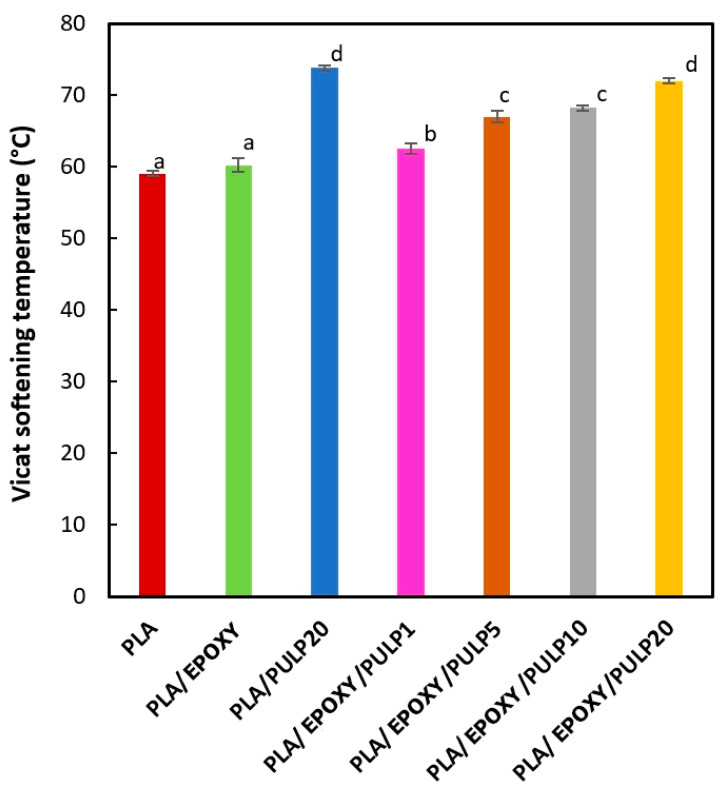
The Vicat softening temperature of PLA, PLA/EPOXY, PLA/PULP, and PLA/EPOXY with 1–20% bamboo pulp content. The mean values indicated by the different lowercase letters significantly differ (*p* < 0.05).

The Vicat softening temperature (VST) values of the PLA, PLA/EPOXY, and PLA/EPOXY/PULP composites are shown in Figure 8. The VST results represent the heat resistance of the composites from the solid to the rubbery states. PLA and PLA/EPOXY showed a VST of 58 °C and 60 °C, respectively. The addition of bamboo pulp increased the VST from 58 °C in pure PLA to 73.8 °C in the PLA/PULP composite. This can be attributed to the nucleating effect of the bamboo pulp, enhancing the crystallinity of PLA, as shown in Table 2. Moreover, the increased crystallinity contributed to the improved thermal stability of the composite, leading to a higher VST value. The VST of PLA/EPOXY/PULP1 is lower owing to PLA crystal obstruction by the EPOXY reaction and a small amount of pulp. However, increasing the bamboo pulp content in the PLA/EPOXY/PULP composites raises the VST, attributed to the enhanced PLA crystallinity facilitated by the nucleating effect of bamboo pulp. The bamboo pulp acts as a nucleating agent, increasing the thermal stability of the composites and resulting in a higher VST.

### 3.7. Contact Angle

**Figure 9:**
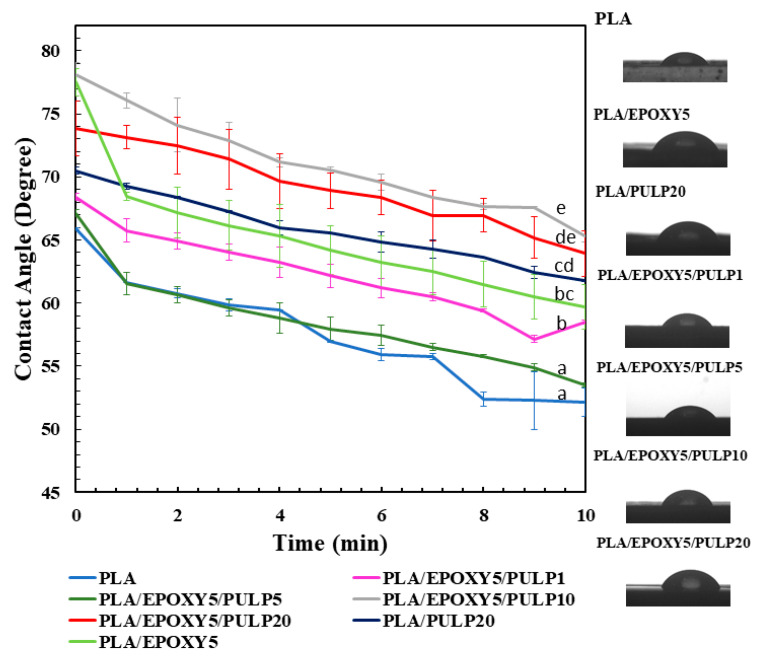
Water droplet contact angle of PLA, PLA/EPOXY, PLA/PULP, and PLA/EPOXY with 1–20% of bamboo pulp. The mean values indicated by the different lowercase letters significantly differ (*p* < 0.05).

Contact-angle measurements were used to evaluate the surface tension of the PLA composites. Figure 9 shows the water droplet contact angles of PLA and the PLA/EPOXY/PULP composites. The water droplet contact angle of the PLA composites decreased with time from 0 to 10 min. After 10 min, the water droplet contact angle of PLA increased from 52° to 63° with the addition of 20% pulp. The introduction of EPOXY/PULP composites (1%, 5%, 10%, and 20%) also resulted in varying contact angles: EPOXY/PULP 1% (61°), EPOXY/PULP 5% (55°), EPOXY/PULP 10% (66°), and EPOXY/PULP 20% (64°). The low water droplet contact angle of PLA was owing to hydrophilicity introduced by the –COOH and –OH end groups of PLA [45]. The water contact angle of PLA increased with the addition of EPOXY, owing to the crosslinking network formed via the EPOXY reaction. Adding PULP 20% to PLA increased the water droplet contact angle because of the rough surface of the pulp. The surface properties of bamboo pulp play a significant role in morphology changes. The presence of lignin, a hydrophobic material, on the surface of bamboo pulp contributes to its hydrophobic nature. The addition of EPOXY to PLA/PULP10 and PLA/PULP20 further increased the contact angle owing to the crosslinking between PLA and the surface of the pulp through the EPOXY reaction, as well as the hydrophobicity of the lignin present in the pulp. The PLA/EPOXY/PULP10 showed a slightly higher contact angle than that of PLA/EPOXY/PULP20 owing to the high amount and hydrophilicity of the fibers in the bamboo pulp. In addition, the crosslinking reaction between PLA and bamboo pulp by EPOXY decreased the space between both surfaces and increased the water resistance of the composites [46]. The lower contact angles of PLA/EPOXY/PULP1 and PLA/EPOXY/PULP5 were attributed to the combined effects of the high hydrophilicity of PLA, low PULP content, large surface gaps, and low interfacial crosslinking between PLA and PULP.

## 4. Conclusions

A PLA/EPOXY/PULP composite was successfully developed by melt-mixing PLA with PULP and EPOXY. FTIR confirmed the reaction between the epoxy groups of EPOXY and the –COOH of PLA, and the –OH groups of PULP. The tensile strength of PLA/EPOXY/PULP20 increased to 76.8 MPa compared to pure PLA (57.5 MPa). The addition of 20% bamboo pulp in PLA/EPOXY resulted in the highest tensile strength owing to the enhanced mechanical properties of bamboo pulp. However, it also led to a decrease in the elongation at break, indicating reduced flexibility, which can be attributed to the reaction facilitated by EPOXY. PLA/EPOXY/PULP5 showed the best improvement of high toughness properties with 764 folding cycles, 68 MPa tensile strength, and 6% elongation at break. EPOXY decreased the space and induced a connection between the PLA and PULP surfaces. Moreover, EPOXY enhanced the interfacial tension between PLA and the PLA/EPOXY/PULP composites via the EPOXY reaction, improving the water resistance of the composite. PULP acts as a nucleating agent to induce crystallinity in PLA, improving the thermal stability of the composite. Furthermore, EPOXY acts as a plasticizer for PLA to decrease T_g_, provide flexibility to the PLA sample, and induce crosslinking inside the PLA and PLA/PULP composites. This reaction enhances the crosslinking inside PLA and the interfacial adhesion between PLA and PULP, improving the tensile strength, toughness, thermal, and water resistance properties of the composites. Notably, the resulting PLA composites have excellent properties and can be used for packaging and agricultural applications.

## Figures and Tables

**Table 1:** Composition and code name of PLA, EPOXY, and PULP composites.

Sample	Composition (wt/wt%)		
	PLA	EPOXY	PULP
PLA	100	-	-
PLA/EPOXY5	95	5	-
PLA/PULP20	80	-	20
PLA/EPOXY5/PULP1	94	5	1
PLA/EPOXY5/PULP5	90	5	5
PLA/EPOXY5/PULP10	85	5	10
PLA/EPOXY5/PULP20	75	5	20

**Table 2:** The DSC data of PLA with different pulp contents and EPOXY 5%.

Sample	T_g_ (°C)	T_c_ (°C)	T_m_ (°C)	ΔH_c_ (J/g)	ΔH_m_ (J/g)	X_c_ (%)
PLA	56	126	163	22.5	31.3	9.4
PLA/EPOXY	51	121	163	32.3	35.5	3.6
PLA/PULP20	56	105	163	19.7	31.0	15.2
PLA/EPOXY/PULP1	51	100	161	29.6	36.1	7.4
PLA/EPOXY/PULP5	51	93	161	15.8	30.2	17.2
PLA/EPOXY/PULP10	51	104	161	18.7	33.0	18.1
PLA/EPOXY/PULP20	51	100	161	15.0	28.1	18.8

## Data Availability

The data presented in this study are available upon request from the corresponding author.

## Abbreviations

DSC, differential scanning calorimetry; FTIR, Fourier-transform infrared spectroscopy; PLA, poly (lactic acid); SEM, scanning electron microscopy; T_c_, crystallization temperature; T_g_, glass transition temperature; T_m_, melting temperature; VST, Vicat softening temperature.

## References

[1] Khalid M.Y., al Rashid A., Arif Z.U., Ahmed W., Arshad H., Zaidi A.A. (2021). Natural fiber reinforced composites: Sustainable materials for emerging applications. Results Eng..

[2] Rahmadiawan D., Abral H., Kotodeli R.A., Sugiarti E., Muslimin A.N., Admi R.I., Arafat A., Kim H.-J., Sapuan S.M., Kosasih E.A. (2023). A Novel highly conductive, transparent, and strong pure-cellulose film from TEMPO-oxidized bacterial cellulose by increasing sonication power. Polymers..

[3] Fattahi Meyabadi T., Dadashian F., Mir Mohamad Sadeghi G., Ebrahimi Zanjani Asl H. (2014). Spherical Cellulose Nanoparticles Preparation from Waste Cotton Using a Green Method. Powder. Technol..

[4] Rahmadiawan D., Abral H., Yesa W.H., Handayani D., Sandrawati N., Sugiarti E., Muslimin A.N., Sapuan S.M., Ilyas R.A. (2022). White Ginger Nanocellulose as Effective Reinforcement and Antimicrobial Polyvinyl Alcohol / ZnO Hybrid Biocomposite Films Additive for Food Packaging Applications. J. Compos. Sci..

[5] Shi S.C., Liu G.T. (2021). Cellulose Nanocrystal Extraction from Rice Straw Using a Chlorine-Free Bleaching Process. Cellulose..

[6] Landes A., Letcher T. (2020). Mechanical strength of bamboo filled PLA composite material in fused filament fabrication. J. Compos. Sci..

[7] Nayak L., Mishra S.P. (2016). Prospect of bamboo as a renewable textile fiber, historical overview, labeling, controversies and regulation. Fash Text..

[8] Shah A.U.M., Sultan M.T.H., Jawaid M., Cardona F., Talib A.R.A. (2016). A Review on the Tensile Properties of Bamboo Fiber Reinforced Polymer Composites. BioResources.

[9] Deshpande A.P., Bhaskar Rao M., Lakshmana Rao C.L. (2000). Extraction of bamboo fibers and their use as reinforcement in polymeric composites. J. Appl. Polym. Sci..

[10] Okubo K., Fujii T., Thostenson E.T. (2009). Multi-scale hybrid bio composite: Processing and mechanical characterization of bamboo fiber reinforced PLA with micro fibrillated cellulose. Compos. A.

[11] Lee S.H., Wang S. (2006). Biodegradable polymers/bamboo fiber bio composite with bio-based coupling agent. Compos. A.

[12] Jantanasakulwong K., Kobayashi Y., Kuboyama K., Ougizawa T. (2016). Thermoplastic Vulcanizate Based on Poly(lactic acid) and Acrylic Rubber Blended with Ethylene Ionomer. J Macromol. Sci. B.

[13] Nofar M.R., Sacligil D., Carreau P.J., Kamal M.R., Heuzey M.C. (2019). Poly (lactic acid) blends: Processing, properties and applications. Int. J. Biol. Macromol..

[14] Kiattipornpithak K., Thajai N., Kanthiya T., Rachtanapun P., Leksawasdi N., Phimolsiripol Y., Rohindra D., Ruksiriwanich W., Sommano S.R., Jantanasakulwong K. (2021). Reaction mechanism and mechanical property improvement of poly(lactic acid) reactive blending with epoxy resin. Polymers.

[15] Kanthiya T., Kiattipornpithak K., Thajai N., Phimolsiripol Y., Rachtanapun P., Thanakkasaranee S., Leksawasdi N., Tanadchangsaeng N., Sawangrat C., Wattanachai P. (2022). Modified poly(lactic acid) epoxy resin using chitosan for reactive blending with epoxidized natural rubber: Analysis of annealing time. Polymers.

[16] Verma C., Olasunkanmi L.O., Akpan E.D., Quraishi M.A., Dagdag O., El Gouri M.E., Sherif E.-S.M., Ebenso E.E. (2020). Epoxy resins as anticorrosive polymeric materials: A review. React. Funct. Polym..

[17] Bucknall C.B., Gilbert A.H. (1989). Toughening tetrafunctional epoxy resins using polyetherimide. Polymer.

[18] Hourston D.J., Lane J.M. (1992). The toughening of epoxy resins with thermoplastics: 1. Trifunctional epoxy resin-polyetherimide blends. Polymer.

[19] Park S.J., Kim H.C. (2001). Thermal stability and toughening of epoxy resin with polysulfone resin. J. Polym. Sci. B Polym. Phys..

[20] Silva I.D.S., Barros J.J.P., Albuquerque A., Jaques N.G., Fook M.V.L., Wellen R.M.R. (2020). Insights into the curing kinetics of epoxy/PLA: Implications of the networking structure. Express Polym. Lett..

[21] Hu L., Vuillaume P.Y. (2020). Chapter 7. Reactive compatibilization of polymer blends by coupling agents and interchange catalysts. Compatibilization of Polymer Blends.

[22] Kodsangma A., Homsaard N., Nadon S., Rachtanapun P., Leksawasdi N., Phimolsiripol Y., Insomphun C., Seesuriyachan P., Chaiyaso T., Jantrawut P. (2020). Effect of sodium benzoate and chlorhexidine gluconate on a biothermoplastic elastomer made from thermoplastic starch-chitosan blended with epoxidized natural rubber. Carbohydr. Polym..

[23] Yan T., Li X., Xu H., Li Y. (2022). Vitrimer-like transparent blend films based on reactive blending of PC and PMMA with catalysis of Mg(TFSI)_2_. Compos. Commun..

[24] Jantanasakulwong K., Wongsuriyasak S., Rachtanapun P., Seesuriyachan P., Chaiyaso T., Leksawasdi N., Techapun C. (2018). Mechanical properties improvement of thermoplastic corn starch and polyethylene-grafted-maleicanhydride blending by Na+ ions neutralization of carboxymethyl cellulose. Int. J. Biol. Macromol..

[25] Thomas R., Yumei D., Yuelong H., Le Y., Moldenaers P., Weimin Y., Czigany T., Thomas S. (2008). Miscibility, morphology, thermal, and mechanical properties of a DGEBA based epoxy resin toughened with a liquid rubber. Polymer.

[26] Limsukon W., Auras R., Selke S. (2019). Hydrolytic degradation and lifetime prediction of poly(lactic acid) modified with a multifunctional epoxy-based chain extender. Polym. Test..

[27] Poongavalappil S., Svoboda P., Theravalappil R., Svobodova D., Vasek V., Jantanasakulwong K., Ougizawa T. (2011). Cross-linking kinetics study and high temperature mechanical properties of ethylene-octene copolymer (EOC)/dicumylperoxide(DCP) system. Eur. Polym. J..

[28] Svoboda P., Svobodova D., Mokrejs P., Vasek V., Jantanasakulwong K., Ougizawa T., Inoue T. (2015). Electron beam crosslinking of ethylene-octene copolymers. Polymer.

[29] Yu X., Wang X., Zhang Z., Peng S., Chen H., Zhao X. (2019). High-performance fully bio-based poly(lactic acid)/polyamide11 (PLA/PA11) blends by reactive blending with multi functionalized epoxy. Polym. Test..

[30] Nguyen V.K., Nguyen T.T., Pham Thi T.H., Pham T.T. (2020). Effects of pulp fiber and epoxidized tung oil content on the properties of biocomposites based on polylactic acid. J. Compos. Sci..

[31] Paunonen S., Berthold F., Immonen K. (2020). Poly(lactic acid)/pulp fiber composites: The effect of fiber surface modification and hydrothermal aging on viscoelastic and strength properties. J. Appl. Polym. Sci..

[32] Immonen K., Anttila U., Wikström L. (2019). Coupling of PLA and bleached softwood kraft pulp (BSKP) for enhanced properties of biocomposites. J. Thermoplast. Compos. Mater..

[33] Chieng B.W., Ibrahim N.A., Yunus W.M.Z.W., Hussein M.Z. (2014). Poly(lactic acid)/poly(ethylene glycol) polymer nanocomposites: Effects of graphene nanoplatelets. Polymers.

[34] Hu G., Cai S., Zhou Y., Zhang N., Ren J. (2018). Enhanced mechanical and thermal properties of poly (lactic acid)/bamboo fiber composites via Surface modification. J. Reinf. Plast. Compos..

[35] Shui T., Chae M., Bressler D.C. (2020). Cross-linking of thermally hydrolyzed specified risk materials with epoxidized poly (vinyl alcohol) for tackifier applications. Coatings.

[36] Coppola B., Cappetti N., Maio L.D., Scarfato P., Incarnato L. (2018). 3D printing of PLA/clay nanocomposites: Influence of printing temperature on printed samples properties. Materials.

[37] Mysiukiewicz O., Barczewski M., Skórczewska K., Matykiewicz D. (2020). Correlation between processing parameters and degradation of different polylactide grades during twin-screw extrusion. Polymers.

[38] Agaliotis E.M., Ake-Concha B.D., May-Pat A., Morales-Arias J.P., Bernal C., Valadez-Gonzalez A., Herrera-Franco P., Proust G., Koh-Dzul J.F., Carrillo J.G. (2022). Tensile behavior of 3D printed polylactic acid (PLA) based composites reinforced with natural Fiber. Polymers.

[39] Bartos A., Nagy K., Anggono J., Antoni, Purwaningsih H., Moczo J., Pukanszky B. (2021). Biobased PLA/sugarcane bagasse fiber composites: Effect of fiber characteristics and interfacial adhesion on properties. Compos. Part A Appl. Sci. Manuf..

[40] Morales A.P., Güemes A., Fernandez-Lopez A., Valero V.C., De S., Llano L.R. (2017). Materials bamboo-polylactic acid (PLA) composite material for structural applications. Materials.

[41] Bhagia S., Lowden R.R., Erdman D., Rodriguez M., Haga B.A., Solano I.R.M., Gallego N.C., Pu Y., Muchero W., Kunc V. (2020). Tensile properties of 3D-printed wood-filled PLA materials using poplar trees. Appl. Mater. Today.

[42] Zhang Q., Shi L., Nie J., Wang H., Yang D. (2012). Study on poly(lactic acid)/natural fibers composites. J. Appl. Polym. Sci..

[43] Si W., Zhang S., He Y., Chen Y.-K. (2021). Tailoring flexibility and dispersity of thermoplastic starch gel by controlling intermolecular structure for improving folding endurance of polylactide. Eur. Polym. J..

[44] Hao M., Wu H., Zhu Z. (2017). In situ reactive interfacial compatibilization of polylactide/sisal fiber bio composites via melt-blending with an epoxy-functionalized terpolymer elastomer. R. Soc. Chem.

[45] Tham C.Y., Abdul Hamid Z.A., Ahmad Z., Ismail H. (2014). Surface modification of poly (lactic acid) (PLA) via alkaline hydrolysis degradation. Adv. Mater. Res..

[46] Li W., He X., Zuo Y., Wang S., Wu Y. (2019). Study on the compatible interface of bamboo fiber/polylactic acid composites by in-situ solid phase grafting. Int. J. Biol. Macromol..

